# Supplementary material for: Building and analyzing metacells in single-cell genomics data
Source: Mol Syst Biol. 2024 May 29;20(7):744–66. doi: 10.1038/s44320-024-00045-6 (PMC11220014; doi:10.1038/s44320-024-00045-6)
Supplement: Supplementary file 1 — Appendix [file 44320_2024_45_MOESM1_ESM.pdf]

Appendix

Building and analyzing metacells in single-cell genomics data

Mariia Bilous[1,2,3,4,5], Léonard Hérault[1,2,3,4,5], Aurélie AG Gabriel[1,2,3,4,5], Matei Teleman[1,2,3,4], David Gfeller[1,2,3,4,*]

[1] Department of Oncology, Ludwig Institute for Cancer Research Lausanne, University of Lausanne, 1011 Lausanne, Switzerland.

[2] Agora Cancer Research Centre, 1011 Lausanne, Switzerland.

[3] Swiss Cancer Center Leman (SCCL), Switzerland.

[4] Swiss Institute of Bioinformatics (SIB), 1015 Lausanne, Switzerland.

[5] These authors contributed equally to this work.

[*] Corresponding author: david.gfeller@unil.ch

Table of contents

Appendix Figure S1. Metacells are robust with respect to the number of nearest neighbors used to build kNN network. – page 2

Appendix Figure S2. Compactness and separation are latent-space dependent and correlated to each other. – page 3

Datasets and methods used throughout the Review. – page 5

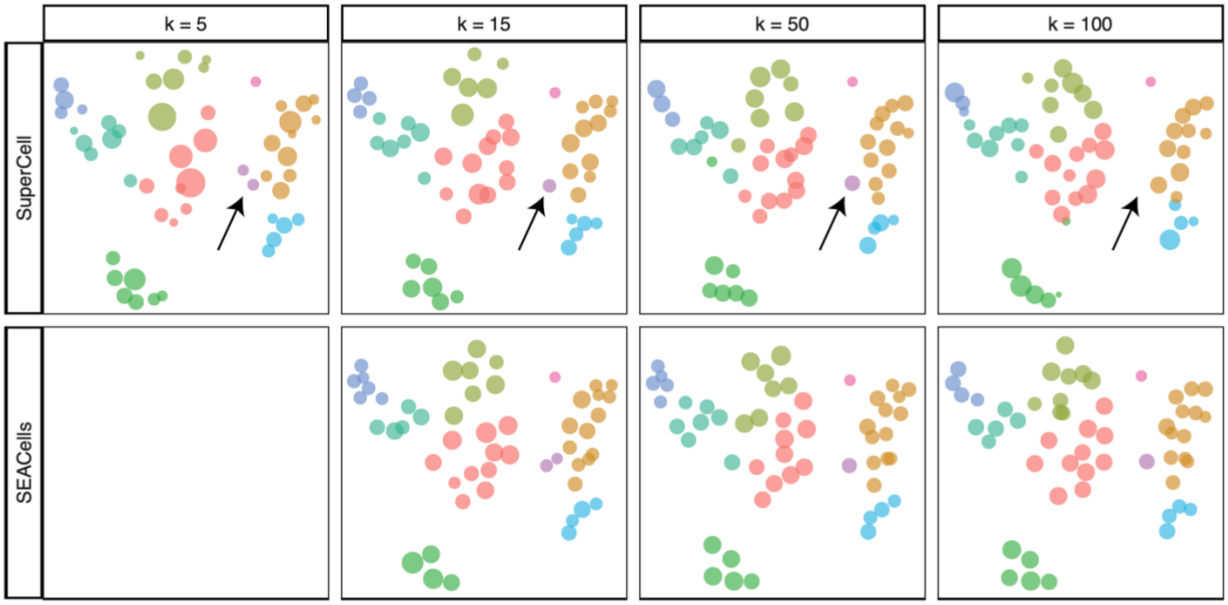

Appendix Figure S1. Metacells are robust with respect to the number of nearest neighbors used to build kNN network.

TSNE of metacells computed for a PBMC dataset with SuperCell (top) or SEACells (bottom) using different number of nearest neighbors k in the kNN network (N=2'700, gamma=50). The coordinates of metacells correspond to the average tSNE coordinates of single cells. Arrows point to the missing cell type at k=100 with SuperCell. SEACells could not be run for k=5 due to memory/time issues.

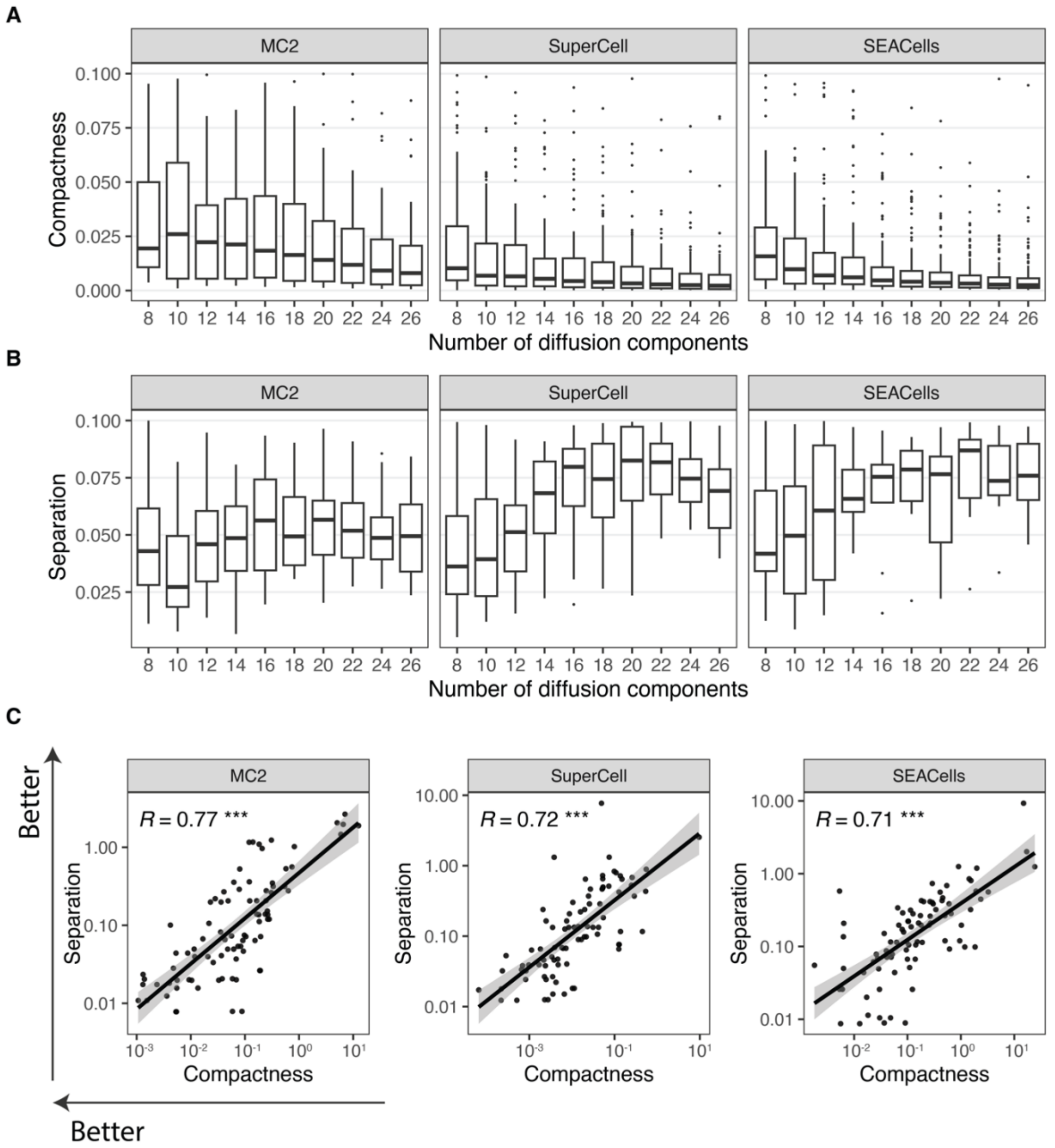

Appendix Figure S2. Compactness and separation are latent-space dependent and correlated to each other.

A Compactness computed at different numbers of diffusion components. B Separation computed at different numbers of diffusion components. C Correlation between metacell compactness and separation. Metacell partition was computed for a PBMC dataset using different metacell

construction tools at a graining level of 30. Compactness and separation were computed in the diffusion component space.

Datasets and methods used throughout the Review

Dataset for illustrating single-cell, metacell, sketching and clustering representations (Fig. 2A; Fig. 3F, Fig. 4; Fig. 7C,D; Fig. 8)

PBMC data from 10X Genomics downloaded from the SeuratData package (Satija Lab, 2020) was used for the majority of illustrative (Fig. 2A; Fig. 3F, Fig. 4; Fig. 7C,D; Fig. 8) and analytical (Fig. 5B; Appendix Fig. S2) purposes. A standard downstream analysis pipeline was applied to obtain a single-cell tSNE (Maaten & Hinton, 2008) representation of data. Cells are colored according to the provided cell-type annotation. Metacell representation was obtained upon applying the SuperCell approach. A 2D representation of metacells was computed by averaging tSNE coordinates of single cells within each metacell (Fig. 2A, Fig. 4, Fig. 7D, Fig. 8). 2D representation of clusters (cell types) was obtained by averaging the tSNE coordinates of single cells within each cluster (Fig. 2A; Fig. 8). The size of metacells, resp. clusters, in tSNE is proportional to the number of single cells in metacells, resp. clusters.

Computing compactness and separation from different latent spaces (Appendix Fig. S2)

To compute compactness and separation for different metacell construction tools from different latent spaces (Appendix Fig. S2), PMBC dataset from 10x Genomics was used as it is, without filtering cells or genes. For MC2 (metacells v.0.8.0), the divide_and_conquer_pipeline() was used with the target_metacell_size being 76’100 UMIs (to obtain the requested graining level of 30). For SuperCell (v.0.1), we used the 10 principal components computed from the top 1’000 variable genes with a graining level of 30. The same PCA embedding and graining level was used for SEACells (v.0.2.0) by requesting 87 metacells, initializing algorithm considering 10 eigenvalues and fitting 25 iterations, with the convergence tolerance of 1e-5. Compactness and separation were computed using the corresponding functions from the SEACells package for different latent spaces (Appendix Fig. S2A,B). Different latent spaces correspond to the diffusion components embedding with different number of dimensions (from 8 to 26).

For the correlation between compactness and separation (Appendix Fig. S2C), the values computed on 10 diffusion components were used.

Correlation between metacell size and number of detected genes (Fig. 5b)

The correlation between the metacell size and the number of detected genes was computed for the PBMC dataset by constructing metacells using SuperCell with a graining level of 10. The metacell size corresponds to the number of single cells in a metacell, the number of detected genes corresponds to the number of genes with at least 1 UMI count in a metacell profile.

Illustrating datasets of different complexity and size (Fig. 2D-G)

To illustrate datasets of different complexity (Fig. 2D), 3 single-cell RNA-seq datasets of the same size were used consisting of T cells, Cord Blood Mononuclear Cells (CBMCs) and Bone Marrow (BM) cells. Filtered and annotated BM (Stuart *et al*, 2019), derived from GEO: GSE128639, and CBMC (Stoeckius *et al*, 2017), derived from GEO:GSE100866. T cells dataset was obtained by selecting cells from the BM dataset annotated to CD4 and CD8 naive and mature cell types. The three datasets were randomly downsampled to 5’000 cells and a standard Seurat pipeline was applied to obtain a UMAP (T, BM datasets) or tSNE (CBMC dataset) representation of the data.

For each dataset we used SuperCell to build metacells at graining levels (gamma) ranging from 1 (single-cells) to 200 in steps of 5 from the 2’000 variable genes identified by Seurat for each dataset. We used 10, 20 and 50 principal components for T, CBMC, and BM dataset respectively. Using single-cell annotation, we annotated each metacell according to the most abundant cell type within it, allowing us to analyze the number of recovered cell type at increasing graining level. A cell type is considered as a recovered one if at least one metacell was annotated to this cell type.

Similarly, we analyzed the influence of the input size of the single cell data on the number of cell types retrieved at increasing graining level. To do this, we use the BM dataset with all 30’000 annotated cells and random subsamples of 5’000 and 1’000 cells.

Benchmarking computational cost of metacell construction tools (Fig. 5C-E)

To evaluate computational cost of different metacell construction tools, we have used the mouse organogenesis atlas (MOCA) data generated by (Cao *et al*, 2019). This atlas contains scRNA-Seq

data from 61 embryos representing a total of around 2 million cells. We first assessed the computational resources (*i.e.* CPU time and memory) needed to construct metacells using SuperCell, SEACells and MC2 on multiple datasets containing an increasing number of embryos (from 3 to 50 embryos, *i.e.* from 10’242 to 900’078 cells) (Fig. 5C). SEACells and SuperCell are limited for datasets larger than 75’000 and 450’000 respectively. However, each algorithm proposes different approaches to accelerate the metacells construction: i) SuperCell can construct metacells for a subset of cells and projects the remaining cells onto the constructed metacells, ii) SEACells proposes to use GPUs to accelerate the process and iii) MC2 can construct the metacells within randomly defined piles using parallel computing. We have used these approaches on the MOCA datasets and show that the computational resources needed decrease significantly. Note that all jobs were run on a machine with 500 GB and time limit of 20 hours with 1 CPU except for the run of MC2 which is able to use multithreading (10 CPUs were used in the latter case). Alternatively, for large datasets containing multiple samples, the metacell construction algorithms can be applied on each sample separately followed by sample integration at the metacell level for downstream analyses (Fig. 5D). We used this approach on the MOCA datasets and show in Fig. 5E that the computational resources needed to build the metacells and to perform standard downstream analyses are substantially lower than those needed for performing similar analyses at the single-cell level. By standard downstream analyses we assume data normalization, feature selection, data scaling, PCA, clustering, UMAP, and differential expression analyses performed with Seurat framework (Hao *et al*, 2023).

Illustrative cartoons (Fig. 1; Fig. 3; Fig. 4, Fig. 5A; Fig. 6; Fig. 7A,B; Fig. 8)

For illustration purposes, no underlying data or real computation were used.

Reference

Cao J, Spielmann M, Qiu X, Huang X, Ibrahim DM, Hill AJ, Zhang F, Mundlos S, Christiansen L, Steemers FJ, *et al* (2019) The single-cell transcriptional landscape of mammalian organogenesis. *Nature* 566: 496–502

Hao Y, Stuart T, Kowalski MH, Choudhary S, Hoffman P, Hartman A, Srivastava A, Molla G, Madad S, Fernandez-Granda C, *et al* (2023) Dictionary learning for integrative, multimodal and scalable single-cell analysis. *Nat Biotechnol*

Maaten L van der & Hinton G (2008) Visualizing Data using t-SNE. *Journal of Machine Learning Research* 9: 2579–2605

Satija Lab (2020) pbmc3k.SeuratData: 3k PBMCs from 10X Genomics. [DATASET]

Stoeckius M, Hafemeister C, Stephenson W, Houck-Loomis B, Chattopadhyay PK, Swerdlow H, Satija R & Smibert P (2017) Simultaneous epitope and transcriptome measurement in single cells. *Nat Methods* 14: 865–868

Stuart T, Butler A, Hoffman P, Hafemeister C, Papalexi E, Mauck WM, Hao Y, Stoeckius M, Smibert P & Satija R (2019) Comprehensive Integration of Single-Cell Data. *Cell* 177: 1888-1902.e21
